# Bone biochemical markers in acromegaly: An association with disease activity and gonadal status

**DOI:** 10.14744/nci.2020.35467

**Published:** 2022-02-10

**Authors:** Mehmet Emin Piskinpasa, Hamide Piskinpasa

**Affiliations:** 1. Department of Internal Medicine, Istanbul Training and Research Hospital, Istanbul, Turkey; 2. Department of Endocrinology and Metabolism, Bakirkoy Dr. Sadi Konuk Training and Research Hospital, Istanbul, Turkey

**Keywords:** Acromegaly, acromegaly disease activity, hypogonadism

## Abstract

**Objective:**

We aim to demonstrate the effect of disease activity and gonadal status on bone biochemical parameters in patients with acromegaly.

**Methods:**

In this cross-sectional,case–control study, 73 patients with acromegaly and 64 healthy controls were included in the study. Acromegaly and control groups, as well as active/controlled acromegaly groupswere compared in terms of alkaline phosphatase (ALP), calcium, magnesium, phosphorus, parathormone (PTH) and 25-OH Vitamin D (25[OH]D), and C-terminal telopeptide of type 1 collagen (CTX). Patients with hypogonadism and normal gonadal status were also compared in terms of these parameters among patients with acromegaly.

**Results:**

The calcium, phosphorus, and CTX were increased in the acromegaly group compared to the control group (p=0.04, p=0.006, and p<0.001, respectively). Age, estimated glomerular filtration rate (eGFR), PTH, and 25(OH)D levels were similar in the acromegaly group and the control group. The ALP, calcium, phosphorus, and CTX were increased in patients with active acromegaly compared to those in remission (p=0.03, p=0.001, p=0.03, and p=0.017, respectively). Age, eGFR, ALP, calcium, and CTX were increased in acromegalic patients with hypogonadism compared in those without hypogonadism (p<0.001, p=0.004, p=0.003, p=0.001, and p=0.009, respectively) while phosphorus, PTH, and 25(OH)D levels were similar between the two groups.

**Conclusion:**

Growth hormone (GH) and insulin-like growth factor 1 (IGF-1) levels, as well as concomitant hypogonadism, play an active role in calcium and CTX levels, while phosphorus levels are associated only with IGF-1 and GH rather than hypogonadism.

**A**cromegaly is a rare disease characterized by increased growth hormone (GH) and insulin-like growth factor 1 (IGF-1). Several comorbidities and metabolic complications are observed in patients with acromegaly due to the effect of increasing GH and IGF-1 levels [1, 2]. GH plays an essential role in the growth, differentiation, and repair of bone and cartilage formation through direct effects and indirect effects through IGF-1 [3]. Despite these physiological effects of GH, the increased GH adversely affects the balance between bone formation and resorption in patients with acromegaly [4, 5]. Although data on the risk of osteoporosis and changes in bone mineral density appear to be contradictory, secondary osteoporosis associated with increased bone turnover has been shown in patients with active acromegaly [5]. Changes in bone mineral levels may also be observed in patients with acromegaly. Hypercalcemia, hyperphosphatemia, and hypercalciuria have been reported in patients with acromegaly [6, 7]. In patients with acromegaly, although there are increases in 1,25-dihydroxyvitamin D (25[OH]D) levels, data on 25(OH)D and parathormone (PTH) are contradictory [7–9].

As with any other complication in acromegalic patients, the change in bone mineral markers has been associated with disease activity, disease duration, concomitant hypogonadism, glucocorticoid overtreatment, and diabetes mellitus [5]. It is known that increased GH and IGF-1 levels play a role in all complications in acromegaly. Changes in bone mineral markers have been observed due to decreased GH and IGF-1 levels through medical and surgical treatment [5].

Hypogonadism may be observed in approximately 50% of patients with acromegaly. Pituitary surgery, concomitant hyperprolactinemia, pituitary radiotherapy, and the mass effect of pituitary adenoma may lead to hypogonadism in these patients [10]. Sex steroids, which decline in the presence of hypogonadism, are known to change bone structure both in males and females. In this situation, hypogonadism is known to affect bone biochemical markers in acromegaly patients.

We aim to demonstrate the effect of disease activity and gonadal status on bone biochemical parameters in patients with acromegaly in this study.

## Materials and Methods

This was a cross-sectional and case–controlled study. Seventy-three patients with acromegaly (46 females/27 males) and 64 healthy controls (41 females/23 males) with normal IGF-1, GH were included in the study. Healthy controls were matched by age ±2 years and body mass index (BMI) ±2 kg/m^2^ to the patients with acromegaly.

The patients with chronic renal impairment estimated glomerular filtration rate (eGFR) <60 mL/min/1.73 m^2^), chronic liver disease, hyperthyroidism, diabetes mellitus, or primary hyperparathyroidism were excluded from the study.

The diagnosis of acromegaly was defined by the presence of typical clinical features, radiographic findings, non-suppressible GH levels, and high IGF-I level, according to the guideline [10]. Acromegaly disease activity at the last visit was evaluated according to “A Consensus on Criteria for Cure of Acromegaly, 2010” [11]. Active disease is defined as high IGF-1 levels according to age-adjusted normal range and random GH level >1 ng/ml (μg/l) and GH >0.4 ng/ml. Criteria of acromegaly remission are defined as IGF-1 level in the age-adjusted normal range, random GH level <1 ng/ml, or GH <0.4 ng/ml after the oral glucose load.

**Highlight key points**

• Changes in bone mineral levels may be observed in patients with acromegaly.
• GH and IGF-1 levels and concomitant hypogonadism play an active role in the increase in calcium, ALP, and CTX levels in patients with acromegaly.
• IGF-1 and GH have more effect on the phosphorus levels in patients with acromegaly rather than hypogonadism.

The normal gonadal function was defined as regular menstrual periods and lack of estrogen deficiency in women and testosterone levels in the normal range by age in men [12–14]. Patients receiving testosterone or estrogen replacement were considered to have a normal gonadal function. BMI was evaluated in all study groups. BMI was calculated as weight (kg)/height (m^2^).

Venous blood samples were drawn in following overnight fasting. Serum glucose, creatinine, alanine aminotransferase (ALT), alkaline phosphatase (ALP), calcium, magnesium, and phosphorus were measured by the photometric method using a Beckman Coulter analyzer. PTH and 25(OH)D levels were measured by chemiluminescence method using a Beckman Coulter Analyzer. C-terminal telopeptide of type 1 collagen (CTX) was measured by electrochemiluminescence method using a Roche Diagnostic Autoanalyzer System.

We calculated corrected calcium level according to albumin level using following formula: [corrected calcium=serum calcium + 0.8 × (4- patient’s albumin)].

Serum IGF-1 level was measured using a solid-phase, enzyme-labeled chemiluminescent enzyme immunometric assay (IMMULITE 2000). Serum GH levels were measured using a two-site chemiluminescent immunometric assay (IMMULITE 2000) with a sensitivity of 0.01 g/l. IGF-1 levels were corrected using the formula “IGF-1 levels/upper limit of normal (ULN)” with respect to the ULN. Accordingly to this, the IGF-1 level was considered as normal in patients with IGF-1 ULN ≤1.

Patients with acromegaly and healthy controls were compared in terms of age, BMI, gender, glucose, creatinine, eGFR, ALT, ALP, calcium, magnesium, phosphorus, PTH, 25(OH)D, and CTX. Active and controlled acromegaly groups were compared in terms of similar parameters.

Patients with hypogonadism and normal gonadal status in the acromegaly group were also compared.

Correlation analyses were performed in terms of ALP, calcium, magnesium, phosphorus, PTH, 25(OH)D, and CTX that may be associated with GH and IGF-1 levels in the whole study group.

The study was approved by the local ethical committee and was conducted according to the Declaration of Helsinki. Informed consent was obtained from all patients with acromegaly and healthy controls.

Our study was approved by the ethics committee in March 2019 and the number was 201,956.

### Statistical Analyses

Statistical analyses were performed using SPSS version 22.0. Categorical variables were defined by frequency and percentage rate, and numerical variables were defined by mean±standard deviation (SD). The normality of the distribution of the quantitative variables was assessed by the Kolmogorov–Smirnov test. Independent group comparisons, one-way ANOVA test was performed for normally distributed numeric variables, and the Kruskal–Wallis test was performed for non-normally distributed data. Categorical variables were compared using the Chi-square test. Spearman correlation analysis was used for correlation analysis. Statistically significant results were defined with p<0.05.

## Results

Age was similar in the acromegaly group and healthy control group in this study (48.1±12.5 and 47.9±13.5 years, respectively). About 56% (n=41) of the patients with acromegaly had the active disease (14 patients with newly diagnosed acromegaly and 27 patients with the active disease under treatment), and 44% (n=32) of the patients were in remission (13 patients had been surgically cured and 19 patients had acromegaly that was well controlled medically). Clinical characteristics and laboratory data of the acromegaly and the control groups are presented in Tables 1 and 2.

**Table 1.** Comparison of control and acromegaly groups in terms of clinical and laboratory findings (n=137)

		Acromegaly group (n=73)	Control group (n=64)	p
Age (years) Mean±SD	48.1±12.5	47.9±13.5	NS
Female (n)/male (n)	46/27	41/23	NS
BMI (kg/m^2^) Mean±SD	29.9±6.0	28.9±5.1	NS
Creatinine (mg/dL) Mean±SD	0.7±0.17	0.73±0.2	NS
eGFR Mean±SD	104.4±16.9	100.5±17.9	NS
ALT Mean±SD	16.7±6.9	27.0±17.6	<0.001
ALP (U/L) Mean±SD	80.5±29.6	83.6±34	NS
Calcium (mg/dL) Mean±SD	9.3±0.54	9.1±0.48	0.04
Phosphorus (mg/dL) Mean±SD	3.8±0.51	3.5±0.45	0.006
Magnesium (mg/dL) Mean±SD	1.8±0.18	2±0.14	<0.001
PTH (pg/mL) Mean±SD	54.5±25.7	50.4±27.3	NS
25(OH)D (ng/mL) Mean±SD	22.1±12	23.5±12	NS
CTX Mean±SD	0.54±0.31	0.34±0.17	<0.001
GH (ng/ml) Mean±SD	4.4±12.9	2.1±0.64	<0.001
IGF-1 ULN Mean±SD	1.3±0.8	0.55±0.16	<0.001

P<0.05 statistically significant, significant p values are shown in bold. SD: Standard deviation; BMI: Body mass index; ALP: Alkaline phosphatase; ALT: Alanine aminotransferase; PTH: Parathyroid hormone; CTX: Cross-linked C-telopeptide of type I collage; eGFR: Estimated glomerular filtration rate; GH: Growth hormone; IGF-1 ULN: Insulin-like growth factor-1 upper limit of normal.

**Table 2.** Clinical and biochemical comparison of acromegalic patients grouped according to disease control (n=137)

	Control group (Group A) (n=64)	Acromegalic patients with active disease (Group B) (n=41)	Acromegalic patients with controlled disease (Group C) (n=32)	p		
				p^1^	p^2^	p^3^
Age (years) Mean±SD	47.9±13.5	49.3±12	46.5±13.1	NS	NS	NS
Female (n)/male (n)	41/23	25/16	21/11	NS	NS	NS
BMI (kg/m^2^) Mean±SD	28.9±5.1	30.5±6.9	29.5±5.2	NS	NS	NS
Creatinine (mg/dL) Mean±SD	0.73±0.2	0.67±0.14	0.73±0.19	NS	NS	NS
eGFR Mean±SD	100.5±17.9	105.7±15.3	102.6±18.8	NS	NS	NS
ALT Mean±SD	27.0±17.6	16.5±6.0	16.9±8.0	0.001	0.001	NS
ALP (U/L) Mean±SD	83.6±34	88.4±31.3	71.7±25.1	NS	NS	0.03
Calcium (mg/dL) Mean±SD	9.1±0.48	9.5±0.55	9.0±0.39	0.001	NS	0.001
Phosphorus (mg/dL) Mean±SD	3.5±0.45	3.9±0.55	3.6±0.41	0.001	NS	0.03
Magnesium (mg/dL) Mean±SD	2±0.14	1.8±0.21	1.9±0.15	<0.001	0.02	NS
PTH (pg/mL) Mean±SD	50.4±27.3	53.9±21.3	55.1±30.3	NS	NS	NS
25(OH)D (ng/mL) Mean±SD	23.5±12	19.2±10	25.7±13.4	NS	NS	NS
CTX Mean±SD	0.34±0.17	0.62±0.35	0.44±0.22	<0.001	NS	0.017
GH (ng/ml) Mean±SD	2.1±0.64	16.8±7.4	1.1±0.7	<0.001	NS	<0.001
IGF-1 ULN Mean±SD	0.55±0.16	1.75±0.82	0.75±0.28	<0.001	0.01	<0.001

P^1^: Difference between Groups A and B; P^2^: Difference between Groups A and C; P^3^: Difference between Groups B and C; P<0.05 statistically significant, significant p values are shown in bold; SD: Standard deviation; BMI: Body mass index; ALP: Alkaline phosphatase; ALT: Alanine aminotransferase; PTH: Parathyroid hormone; CTX: Cross-linked C-telopeptide of type I collage; GH: Growth hormone; IGF-1 ULN: Insulin-like growth factor-1 upper limit of normal.

Age was more advanced in patients with hypogonadism than the patients without hypogonadism (54±11.8 and 43±11.2 years, respectively). Hypogonadism was present in 42% (n=31) of the patients diagnosed with acromegaly. Of those with hypogonadism (n=31), 26 were male, and 5 were female. Seventeen female patients with hypogonadism were also postmenopausal. The group without hypogonadism (n=42) consisted of 20 female and 22 male patients. Clinical characteristics and laboratory data of the patients with and without hypogonadism are presented in Table 3.

**Table 3.** Clinical and biochemical comparison of acromegalic patients grouped according to gonadal status (n=73)

	Acromegalic patients with hypogonadal status (n=31)	Acromegalic patients with normal gonadal status (n=42)	p
Age (years) Mean±SD	54±11.8	43±11.2	<0.001
Female (n)/male (n)	26/5	20/22	<0.003
BMI (kg/m^2^) Mean±SD	31.3±7.2	29.2±5.2	NS
Active disease (n)/controlled disease (n)	10/21	20/22	NS
Creatinine (mg/dL) Mean±SD	0.6±0.16	0.7±0.17	NS
eGFR Mean±SD	99.3±17.9	108.2±15.1	0.004
ALT Mean±SD	16.9±7.1	16.5±6.9	NS
ALP (U/L) Mean±SD	92.5±34.5	71.2±21.1	0.003
Calcium (mg/dL) Mean±SD	9.5±0.52	9.1±0.48	0.001
Phosphorus (mg/dL) Mean±SD	3.9±0.56	3.7±0.47	NS
Magnesium (mg/dL) Mean±SD	1.8±0.18	1.9±0.17	NS
PTH (pg/mL) Mean±SD	60.6±31.3	49.9±19.8	NS
25(OH)D (ng/mL) Mean±SD	21.7±10.8	22.4±13	NS
CTX Mean±SD	0.67±0.38	0.44±0.19	0.009
GH (ng/ml) Mean±SD	3±4.3	5.4±16.5	NS
IGF-1 ULN Mean±SD	1.4±0.82	1.2±0.79	NS

P<0.05 statistically significant, significant p values are shown in bold. BMI: Body mass index; ALP: Alkaline phosphatase; ALT: Alanine aminotransferase; PTH: Parathyroid hormone; CTX: Cross-linked C-telopeptide of type I collage; eGFR: Estimated glomerular filtration rate; GH: Growth hormone; IGF-1 ULN: Insulin-like growth factor-1 upper limit of normal.

Table 1 shows the clinical characteristics and laboratory data of the acromegaly and the control groups. As expected, IGF-1-ULN and GH were increased in the group of patients with acromegaly (p<0.001 and p<0.001, respectively). Calcium, phosphorus, and CTX were increased in the acromegaly group compared to the control group (p=0.04, P=0.006, and p<0.001, respectively). On the other hand, magnesium and ALT levels were higher in the control group than the acromegaly group (p<0.001 and p<0.001, respectively). Age, gender, BMI, creatinine, eGFR, PTH, and 25(OH)D levels were similar in the acromegaly group and the control group.

Clinical characteristics and laboratory data of the patients with active acromegaly, those in remission, and the control group are presented in Table 2. As expected, IGF-1-ULN and GH were increased in patients with active acromegaly than those in remission (p<0.001 and p<0.001, respectively). ALP, calcium, phosphorus, and CTX were increased in patients with active acromegaly compared to those in remission (p=0.03, p<0.001, p=0.03, and p=0.017, respectively). Age, gender, BMI, creatinine, eGFR, ALT, magnesium, PTH, and 25(OH)D levels were similar in patients with active acromegaly and those in remission. Furthermore, in subgroup analyses, ALP, calcium, phosphorus, PTH, 25(OH)D, and CTX levels were observed similarities between the healthy control group and in patients in remission.

Clinical characteristics and laboratory data of acromegaly patients with hypogonadism and acromegaly patients without hypogonadism are shown in Table 3. IGF-1-ULN and GH were similar in these two groups. Age, gender, eGFR, ALP, calcium, and CTX were increased in acromegalic patients with hypogonadism compared to acromegalic patients without hypogonadism (p<0.001, p<0.003, p=0.004, p=0.003, p=0.001, and p=0.009, respectively). The two groups were similar in terms of BMI, active disease/controlled disease (active disease %), creatinine, ALT, phosphorus, magnesium, PTH, and 25(OH)D levels.

The correlations between serum IGF-1 ULN, GH, and laboratory parameters in all of the participants are given in Table 4. There were statistically significant positive correlations of GH between calcium, phosphorus, and CTX levels (r=0.257, P=0.005; r=0.461, p<0.001; and r=0.389, p<0.001, respectively) but there were negative correlations of between 25(OH)D levels (r=–0.211, p=0.026, respectively). There were no statistically significant correlations of GH with magnesium, PTH. There were statistically significant positive correlations of IGF1-ULN between calcium, phosphorus, and CTX (r=0.321, p=0.002; r=0.444, p<0.001; and r=0.454, p<0.001, respectively) but there were negative correlations of between magnesium (r=–0.264, p=0.009, respectively). There were no statistically significant correlations of IGF 1-ULN with PTH, 25(OH)D levels.

**Table 4.** Correlation between GH and IGF-1 levels and bone biochemical markers in whole study group (n=137)

	GH		IGF-1 ULN	
	r	p	r	p
ALP (U/L)	0.085	0.321	0.077	0.404
Calcium (mg/dL)	0.257	0.005	0.321	0.002
Phosphorus (mg/dL)	0.461	<0.001	0.444	<0.001
Magnesium (mg/dL)	–0.183	0.085	–0.264	0.009
PTH (pg/mL)	0.045	0.634	–0.042	0.634
25(OH)D (ng/mL)	–0.211	0.026	–0.044	0.626
CTX	0.389	<0.001	0.454	<0.001

P<0.05 statistically significant, significant p values are shown in bold. GH: Growth hormone; IGF-1 ULN: Insulin-like growth factor-1 upper limit of normal; ALP: Alkaline phosphatase; PTH: Parathyroid hormone; CTX: Cross-linked C-telopeptide of type I collage.

## Discussion

This study investigated the association of bone biochemical markers with disease activity and concomitant hypogonadism in patients with acromegaly. As expected, serum calcium, phosphorus, and CTX levels were found statistically significantly higher in patients with acromegaly compared to healthy controls. On the other way, magnesium levels were lower in acromegaly patients than that in healthy controls. Calcium, phosphorus, ALP, and CTX were higher in a patient with active acromegaly compared to those in remission with surgical or medical treatment. In acromegalic patients with hypogonadism, a statistically significant increase was found in calcium, ALP, and CTX levels, while other parameters were similar compared to acromegalic patients without hypogonadism. However, in patients with acromegaly, although phosphorus level was found to increase with disease activity, it was similar in those with and without hypogonadism. PTH and 25(OH)D levels were similar between patients with active disease/controlled disease and patients with hypogonadism/normal gonadal status.

The association of acromegaly with PTH and 25(OH)D levels remains unclear. In patients with acromegaly, no difference in 25(OH)D levels was reported in comparison to healthy subjects [15, 16]. In the literature, increased PTH levels have been observed with long-term somatostatin analog treatment [17]. This situation has been explained with associated secondary hyperparathyroidism due to decreased calcium absorption in patients with acromegaly [17, 18]. In the present study, 25(OH)D and PTH levels were similar between acromegaly patients and healthy controls, as in between patients with active disease and patients with controlled disease. Taken together all these results, it is shown that the acromegaly disease itself and the activity of this disease have no effect on PTH and 25(OH)D levels.

While calcium and phosphorus levels were higher in patients with acromegaly than in healthy controls, as in the literature, we found similar PTH and 25(OH)D levels in these groups [15, 16]. Hence, there must be other contributing factors to changing the bone mineral markers in patients with acromegaly. This finding suggests that the increase in calcium and phosphorus levels may be associated with increased IGF-1 and GH, additionally to PTH. It is known that increased GH and IGF-1 levels affect bone turnover in patients with acromegaly [19]. Therefore, changes in bone structure and bone turnover markers are expected in acromegalic patients. Mild increases in calcium and phosphorus levels are common in patients with acromegaly. Increased extracellular volume is observed in patients with acromegaly, and the tubular defect caused by this increase is thought to result in altered excretion of calcium and phosphorus [20, 21]. In patients with acromegaly, GH is thought to increase hypercalciuria and hyperphosphatemia also in the presence of increased bone turnover due to the effect of IGF-1 [17]. Increased serum phosphate levels have been achieved by increasing phosphate excretion with GH treatment in patients with GH deficiency [22]. This suggests the effect of GH on bone phosphorus levels. Although increases in calcium levels are expected in patients with acromegaly, severe hypercalcemia requiring treatment may also develop due to increased 1,25(OH)2D levels or concomitant primary hyperparathyroidism. Treatment of acromegaly has been shown to decrease hypercalcemia [7, 23].

Furthermore, exogenous GH therapy has been shown to increase 1,25(OH)2D levels in patients with GH deficiency [24]. However, some studies have reported decreased calcium and phosphorus with acromegaly treatment without any change in 1,25(OH)2D levels [25]. Changes in calcium and phosphorus levels have been associated with increased GH and IGF in acromegalic patients, and higher levels have been reported in active patients compared to those with the controlled disease [26].

In our study, calcium, phosphorus, IGF-1-ULN, and GH levels were higher in patients with active acromegaly compared to those in controlled disease activity with medical or surgical treatment. We also found that IGF-1-ULN was higher in patients with controlled acromegaly compared to the healthy controls, while GH levels were similar between these groups. ALP, calcium, phosphorus, PTH, 25(OH)D, and CTX levels were also observed similar between these groups. These findings suggest an effect of increased IGF-1, as well as increased GH, had a primary effect on increased bone biochemical markers. In the correlation analyses, we performed in the whole study group, we identified positive correlations between calcium and phosphorus levels with IGF-1 and GH.

CTX is a bone turnover marker released during bone resorption [27]. This parameter is used for early response to osteoporosis treatment before bone mineral density evaluation. Studies have shown increased CTX levels in patients with acromegaly [28]. Consistent with the literature, CTX levels were higher in acromegaly patients than healthy controls in the present study. Studies in patients with active acromegaly have reported a significant decrease in CTX levels in patients who achieve remission with surgical or medical treatment [16, 29]. In our study, CTX levels were higher in patients with active acromegaly than patients in remission. Considering the active role, GH and IGF play in bone structure changes, the correlation analysis revealed that CTX levels were correlated with IGF-1 and GH levels in the present study. Similar to our study, there are other studies in the literature that report a correlation between CTX and GH and IGF-1 level [30].

Hypogonadism is observed in approximately 50% of patients with acromegaly. While often reversible, it may develop due to concomitant hyperprolactinemia, the effect of a pituitary mass, and pituitary surgery [10]. Hypogonadism was detected in 42% of the patients included in the present study. Calcium, ALP, and CTX levels were higher in patients with hypogonadism compared to those without hypogonadism. On the other hand, 25(OH)D, parathyroid hormone, and phosphorus levels were similar. The vast majority of patients in the acromegaly group with hypogonadism were those in remission. Hence, we believe that hypogonadism alone is effective on the increase in calcium, CTX, and ALP levels. Consistent with our study, there are studies in the literature that report increased calcium and ALP without the difference in phosphorus levels in acromegalic women with hypogonadism [31]. We mentioned before that increased phosphorus has been associated with increased GH and IGF-1 in patients with acromegaly. In the present study, phosphorus levels were similar in patients with and without hypogonadism, and also GH and IGF-1 levels were also similar between these two groups. All these findings suggest that the changes in phosphorus levels may be more associated with increased GH and IGF-1 rather than hypogonadism.

Our study had certain limitations. The study design was retrospective, and we were unable to evaluate the duration of hypogonadism. The patient with hypogonadism was a heterogeneous group consisting of males, postmenopausal women, and premenopausal women. Patients with hypogonadism were older than those without hypogonadism. It is known that bone mineral metabolism changes due to dietary changes, restricted physical activity, and increased Vitamin D deficiency in the elderly. Higher PTH levels are observed in the elderly compared to younger individuals [32, 33]. These findings showed that our study results might have affected in patients with hypogonadism.

### Conclusion

This study revealed that GH and IGF-1 levels, as well as concomitant hypogonadism, play an active role in the increase in calcium, ALP, and CTX levels in patients with acromegaly. However, IGF-1 and GH have more effect on the phosphorus levels in patients with acromegaly rather than hypogonadism.
